# Physiological and Transcriptomic Analyses Demonstrate the Ca^2+^-Mediated Alleviation of Salt Stress in *Magnolia wufengensis*

**DOI:** 10.3390/plants13172418

**Published:** 2024-08-29

**Authors:** Xiuting Zhao, Zhonglong Zhu, Ziyang Sang, Luyi Ma, Qun Yin, Zhongkui Jia

**Affiliations:** 1 State Key Laboratory of Efficient Production of Forest Resources, College of Forestry, Beijing Forestry University, Beijing 100083, China; zhaoxt0906@163.com (X.Z.);; 2 *Magnolia wufengensis* Research Center, Beijing Forestry University, Beijing 100083, China; 3 College of Agriculture, HuBei Three Gorges Polytechnic, Yichang 443199, China; 4 Forest Science Research Institute of Wufeng Tujia Autonomous County, Yichang 443400, China

**Keywords:** salt stress, calcium, transcriptome, *Magnolia wufengensis*

## Abstract

*Magnolia wufengensis*, a newly discovered ornamental species in the Magnoliaceae family, is susceptible to salinity. Moreover, Ca^2+^ is an essential element for plant growth and is receiving increasing attention for its ability to mitigate the negative effects of environmental stress on plants. In the present study, we investigated the effect of Ca^2+^ on the growth and transcriptome of *M. wufengensis* under salt stress. The treatments used here were as follows: control, NaCl (150 mmol/L), CaCl_2_ (5 mmol/L), and NaCl (150 mmol/L) + CaCl_2_ (5 mmol/L). After a 60-day treatment period, plant growth indices were determined, and leaves were collected for physiological analysis and transcriptome investigation. The combined application of NaCl and CaCl_2_ alleviated phenotypic damage and restored seedling growth. Moreover, RNA sequencing data revealed that in the Na vs. control group and the NaCa vs. Na group, there were 968 and 2632 differentially expressed genes, respectively, which were both primarily enriched in secondary metabolism, glutathione metabolism, signaling hormone metabolism, glucose metabolism, and amino acid metabolism. These pathways were analyzed to screen key genes: the adenosine triphosphate (ATP)-binding cassette efflux transporter G1 (ABCG1) genes, which are related to transmembrane transport; the calmodulin genes, which are related to signal transmission; and the glutathione S-transferase (GST), glutathione peroxidase (GPX), and peroxidase (POD) genes related to antioxidant enzymes. Lastly, we constructed a hypothesis model of Ca^2+^-enhanced salt tolerance in *M. wufengensis*. This study reveals the potential mechanisms by which Ca^2+^ enhances the salt tolerance of *M. wufengensis* and provides a theoretical reference for its cultivation in saline areas.

## 1. Introduction

Soil salinization is regarded as one of the most extreme environmental pressures in the world. Approximately 7% of the world’s total land area, approximately 954 million hectares, is affected by salinity [1]. Soil salinization caused by climate change and improper irrigation has been constantly escalating because of industrial development and urbanization in recent years, which limits the sustainability of the ecological environment and the health of global agriculture and forestry [2].

Salinity threatens plant survival, resulting in stunted growth, reduced production, or even plant death. The primary way through which NaCl damages plants is by causing osmotic stress, which is the outflow of water from plant roots due to the high osmotic potential generated by the high concentration of Na^+^ in saline soil [3]. Na^+^-induced oxidative stress results in an excessive accumulation of reactive oxygen species (ROS), which in turn causes membrane lipid peroxidation and membrane system breakdown [4]. When too much Na^+^ from saline soil enters plant cells, intracellular ion imbalance, protein degeneration, and physiological and metabolic disorders occur, which seriously affect the growth and development of plants and even lead to plant death [5,6]. Therefore, plants need strategies to activate physiological and biochemical processes, including ion transport, redox, signal transduction, and metabolic pathways, to improve their salt tolerance [7]. According to previous studies, plants improve their tolerance to salt by removing ROS through increased antioxidant enzyme activities and nonenzymatic antioxidant contents [4]. Moreover, plant tolerance to stress can increase by altering the activity of plant hormones to transmit stress signals [8]. In addition, secondary metabolites such as flavonoids, phenols, terpenes, and alkaloids are also responsible for coping with abiotic stress [9].

Calcium, which is known as the “total regulator of plant cell metabolism”, is an essential element for plant growth and development and participates in every stage of the process, from seed germination to growth and differentiation, morphogenesis, blossoming, and fruit production [10]. Ca combines with the intracellular messenger calmodulin (CaM) to participate in the perception, transmission, response, and expression of stress signals, thereby regulating physiological metabolism and improving plant stress resistance [11]. Exogenous Ca^2+^ increases the tolerance of plants to NaCl. The appropriate addition of CaCl_2_ under salt stress can increase the seed germination rate, improve plant growth, optimize nutritional status, and enhance photosynthetic capacity [12,13]. Other studies have shown that Ca^2+^ is a membrane protector that acts intracellularly under salt stress and can reduce plasma membrane permeability by eliminating ROS [14,15]. However, the exact biochemical pathways and RNA mechanisms through which Ca^2+^ is used to mitigate plant salt stress are still unknown. Currently, the effects of Ca^2+^ on salt stress have mostly been reported in herbaceous plants, such as tomatoes (*Solanum lycopersicum* L.), rice (*Oryza sativa* L.), and *Iris pseudacorus* L., but the ways in which Ca^2+^ affects Magnoliaceae plants such as *M. wufengensis* have not been reported [14,15,16].

*Magnolia wufengensis*, a new species of the Magnoliaceae family discovered in China in 2004, has great ornamental and research value because of its straight trunk, beautiful crown, and rich variation in flower color and shape [17,18]. *M. wufengensis* has been introduced to many cities in China in recent years. Nevertheless, its growth and development are adversely affected when it is introduced to saline areas (based on unpublished research by our research team). We first used different concentration gradients of NaCl to treat *M. wufengensis*, *Yulania biondii*, and *Yulania denudata* in order to explore the salt stress threshold of *M. wufengensis* and the difference in salt tolerance between *M. wufengensis* and other Magnoliaceae species [19]. Then, through a literature review and preliminary experiments, Ca^2+^ was chosen to improve the salt tolerance of *M. wufengensis*. However, whether Ca^2+^ can improve the salt tolerance of *M. wufengensis* and its physiological and molecular mechanisms remain unknown. Based on these limitations, we designed this study to explore this plant’s Ca^2+^-mediated molecular mechanisms, gene expression patterns, and physiological profile through physiological and biochemical analyses, transcriptome sequencing, and comparative transcriptomic analysis. This study will contribute to our understanding of the mechanisms by which Ca^2+^ enhances salt tolerance and will provide a theoretical basis for the transplantation and field management of *M. wufengensis* in saline soil.

## 2. Results

### 2.1. Ca^2+^ Alleviated the Symptoms of Salt Damage in M. wufengensis under Salt Stress

**Figure 1:**
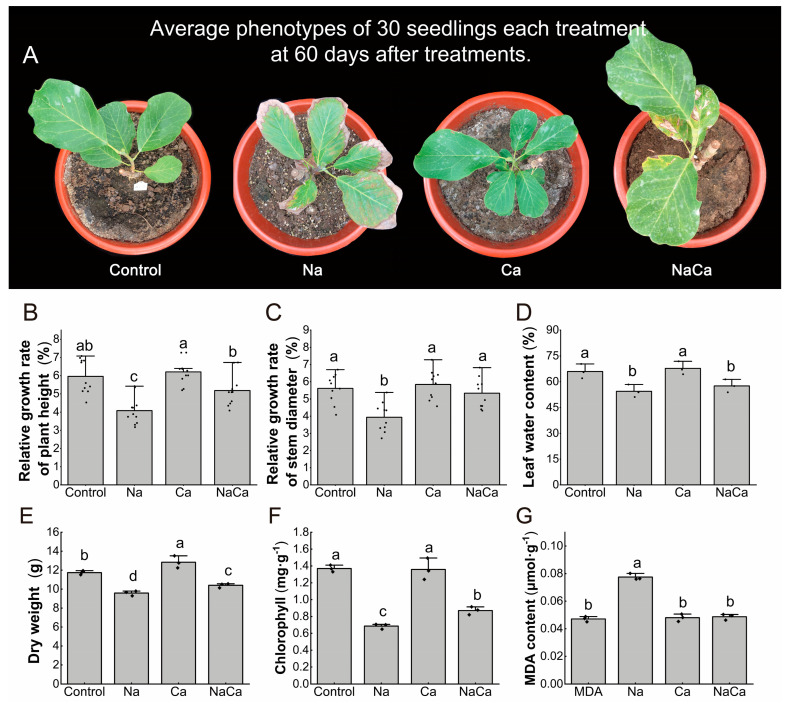
Growth indicators of *M. wufengensis*. (**A**) Phenotypes; (**B**) relative growth rate of plant height; (**C**) relative growth rate of stem diameter; (**D**) leaf water content (LWC); (**E**) dry weight; (**F**) chlorophyll content; and (**G**) MDA content. The statistical data are presented as their means ± SDs. Different lowercase letters in the figure indicate significant differences (*p* < 0.05 according to Duncan’s multiple-comparisons test) among treatments.

NaCl and CaCl_2_ were added to *M. wufengensis* to determine the effect of Ca^2+^ on salt tolerance. Seedlings treated with Ca^2+^ exhibited a healthy phenotype after 60 days, while the leaves of *M. wufengensis* were shriveled and damaged when they were subjected to salt stress. Only the bottom leaves of the seedlings were slightly damaged after the addition of Ca^2+^ to the Na^+^ treatment, and the symptoms of salt damage were significantly lower than those following Na^+^ treatment (Figure 1A). Additionally, we measured the relative growth rate of the height, the relative growth rate of the ground diameter, and the leaf water content of *M. wufengensis* plants under the Na^+^ and Ca^2+^ treatments (Figure 1B–D). Compared with those under normal growth conditions, the growth of *M. wufengensis* plants under salt stress was inhibited. As shown in Figure 1, the height, stem growth, and leaf water content decreased by 29.90%, 29.79%, and 17.50%, respectively. Ca^2+^ alleviated the salt-mediated inhibition of *M. wufengensis* seedling growth. These growth indices did not significantly change under Ca^2+^ treatment compared with the control. In the NaCa treatment, the height-relative growth rate and ground diameter-relative growth rate were significantly greater than those in the salt treatment and were not significantly different from those in the control. Meanwhile, the water content of the leaves was not affected by the addition of Ca^2+^ under the Na treatment or under the control treatment.

Under NaCl stress, the dry weight of the *M. wufengensis* seedlings decreased significantly (Figure 1E), with them weighing only 9.58 ± 0.26 g. Their dry weight increased significantly with the addition of Ca^2+^. Under Ca^2+^ treatment, the dry weight was the highest, reaching 12.84 ± 0.64 g. Under NaCa treatment, the dry weight was lower than that under the control treatment, but significantly higher than that under NaCl stress. Under NaCl stress, the chlorophyll content in the *M. wufengensis* leaves was only 49.89% of that in the control leaves (Figure 1F). The chlorophyll content did not differ significantly with Ca^2+^ addition alone, but was significantly improved under the NaCa treatment. Under NaCl stress, the MDA content was increased by 64.47% to 0.078 ± 0.002 μmol·g^−1^. Meanwhile, after the addition of Ca^2+^, the MDA content decreased, and there was no significant difference compared to the control (Figure 1G).

### 2.2. Sequencing and De Novo Transcriptome Assembly

To reveal the molecular mechanisms by which Ca^2+^ enhances the salt tolerance of *M. wufengensis*, we analyzed the transcriptomes of plants subjected to the control, Na, Ca, and NaCa treatments. We performed three replicates, for a total of twelve samples for RNA extraction. The summary statistics of the clean reads are shown in Table 1. After quality control, an average of 55,848,744 reads were obtained per sample, and 55,358,559 clean reads were obtained. The Q20 value was greater than 97.99% for all of the samples, and the Q30 value was greater than 93.80%. The base error rate was 0.025%, and the GC content was 47.81%, indicating that the sequencing data of the 12 samples in this study are reliable and can be used for follow-up analyses.

**Figure 2:**
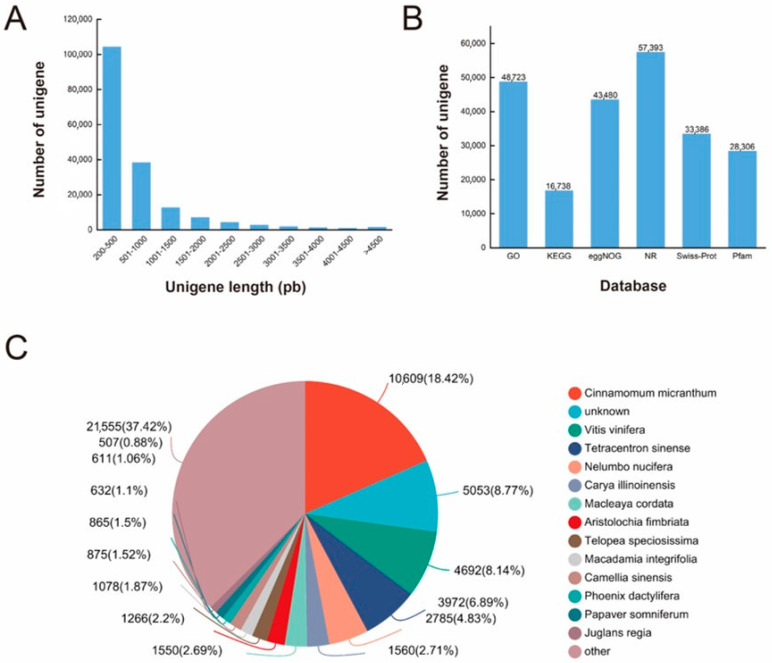
Characteristics of unigenes. (**A**) Distribution of unigene lengths in *M. wufengensis*; (**B**) annotation statistics of *M. wufengensis* unigenes; (**C**) species distribution of the top BLAST hits for each unigene.

All clean reads were assembled using Trinity, as shown in Table 2. A total of 173,292 unigenes were obtained after assembly; the GC percentage was 42.04%, and the total length was 123,213,932 bp, among which the maximum length was 15,917 bp, the minimum length was 201 bp, and the average length was 711 bp. All of the unigenes were sorted from short to long, as shown in Figure 2A and Table S2. There were 104,158 unigenes within the 200–500 bp region, accounting for 60% of the total number of unigenes. There were 38,160 unigenes within the 501–1000 bp region, accounting for 22% of the total unigenes. The total number of unigenes with a length of greater than 1001 bp was 24,127, accounting for 18% of the total. These results demonstrate the accuracy of our transcriptomic results.

### 2.3. Annotation and Classification of M. wufengensis Unigenes

To identify the functions of the *M. wufengensis* unigenes, all of the unigenes were compared with the six major functional databases for annotation (Figure 2B and Table S3). A total of 58,796 genes had known proteins in at least one database. Among them, 16,779 genes were annotated in the Kyoto Encyclopedia of Genes and Genomes (KEGG) database (9.68%), 48,900 genes were annotated in the Gene Ontology (GO) database (28.22%), 43,606 genes were annotated in the Evolutionary Genealogy of Genes: Non-Supervised Orthologous Groups (eggNOG) database (25.16%), 57,610 genes were annotated in the Non-Redundant Protein Sequence (NR) database (33.24%), 33,461 genes were annotated in the Swiss-Prot database (19.31%), and 28,369 genes were annotated in the Pfam database (16.37%).

The unigenes were compared with those in the NR database in order to annotate the proportions of different species statistically (Figure 2C). The results showed that the proportion of *Cinnamomum micranthum* was the highest, accounting for 18.42% of the total number of annotations. The percentages of unigenes in *Vitis vinifera*, *Tetracentron sinense*, *Nelumbo nucifera*, and *Carya illinoinensis* were also relatively high, accounting for 8.14%, 6.89%, 4.83%, and 2.71%, respectively. Unigenes annotated for other species accounted for 37.42% of the total annotated genes.

A total of 208,513 unigenes in the GO database were annotated into 53 categories, which were divided into three main categories: molecular functions (MFs), biological processes (BPs), and cellular components (CCs) (Figure S1A). Among them, “binding” and “catalytic activity” in the MF category, “cellular process” and “metabolic process” in the BP category, and “cell part” and “membrane part” in the CC category were the most abundant. A total of 16,738 unigenes were annotated in the KEGG database, among which “carbohydrate metabolism”, “environmental adaptation”, “transport and catabolism”, “signal transduction”, and “membrane transport” were the most abundant (Figure S1B).

### 2.4. Identification of Differentially Expressed Genes (DEGs)

**Figure 3:**
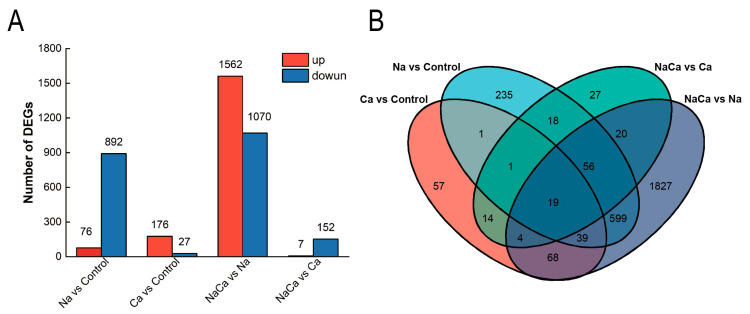
Identification and enrichment analysis of DEGs. (**A**) Number of all DEGs in the four treatment groups; (**B**) Venn diagram showing the numbers of exclusive and common DEGs for the four comparison combinations.

To obtain a comprehensive understanding of gene expression in *M. wufengensis* under salt stress and Ca^2+^ treatment, we identified the DEGs associated with the four treatments (Figure 3A and Table S4). There were 2632 DEGs in the NaCa vs. Na group, of which 1562 were upregulated and 1070 were downregulated, indicating that the addition of Ca^2+^ induced gene expression under salt stress, which may be related to the enhancement of salt tolerance. There were 968 DEGs in the Na vs. control group, 76 of which were upregulated and 892 of which were downregulated. Furthermore, we identified the responsive DEGs that were specific to the Na and Ca treatments using a Venn diagram, which revealed 1827 specific DEGs in the NaCa vs. Na group and 235 DEGs in the Na vs. control group (Figure 3B).

### 2.5. Gene Ontology (GO) and Kyoto Encyclopedia of Genes and Genomes (KEGG) Enrichment Analyses of DEGs

To determine the potential mechanism by which Ca^2+^ enhances the salt tolerance of *M. wufengensis*, we performed GO and KEGG enrichment analyses of the DEGs in the Na vs. control and NaCa vs. Na groups. The data related to the GO and KEGG enrichment analyses of all groups are shown in Tables S5 and S6.

**Figure 4:**
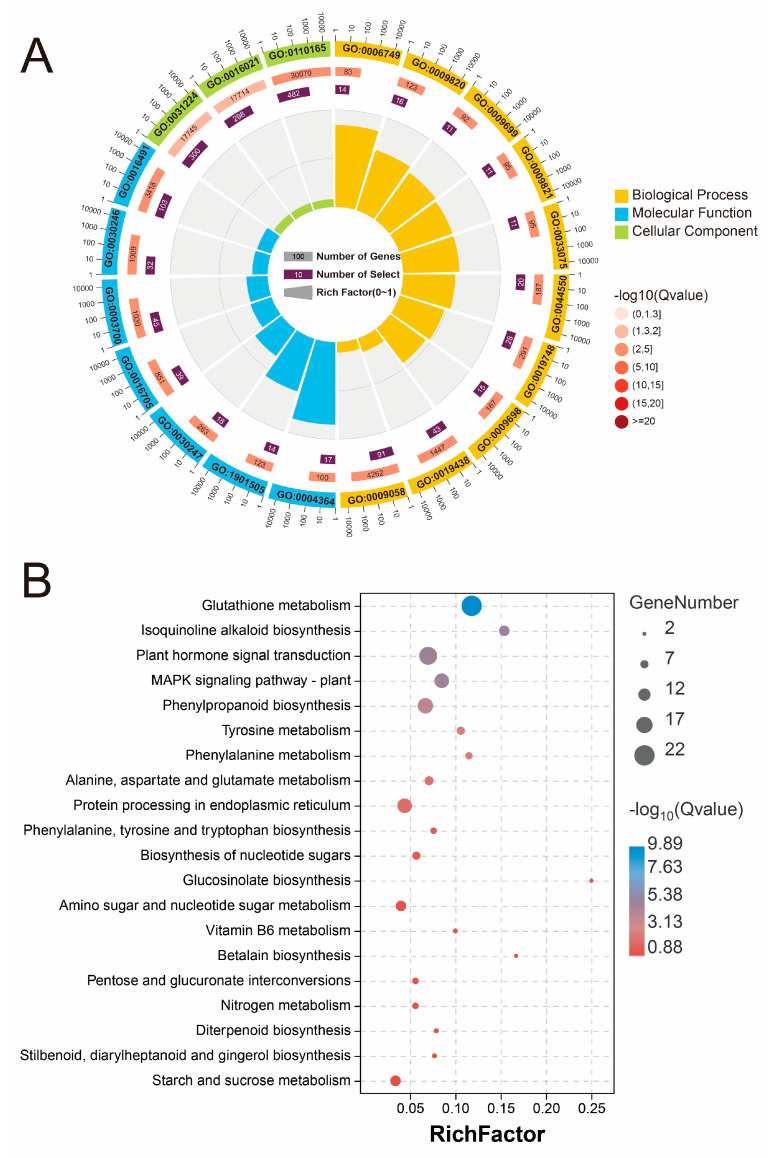
GO and KEGG enrichment analyses of DEGs in the Na vs. control treatment group. (**A**) GO enrichment circle plot of the DEGs; (**B**) KEGG enrichment bubble chart of the DEGs.

**Figure 5:**
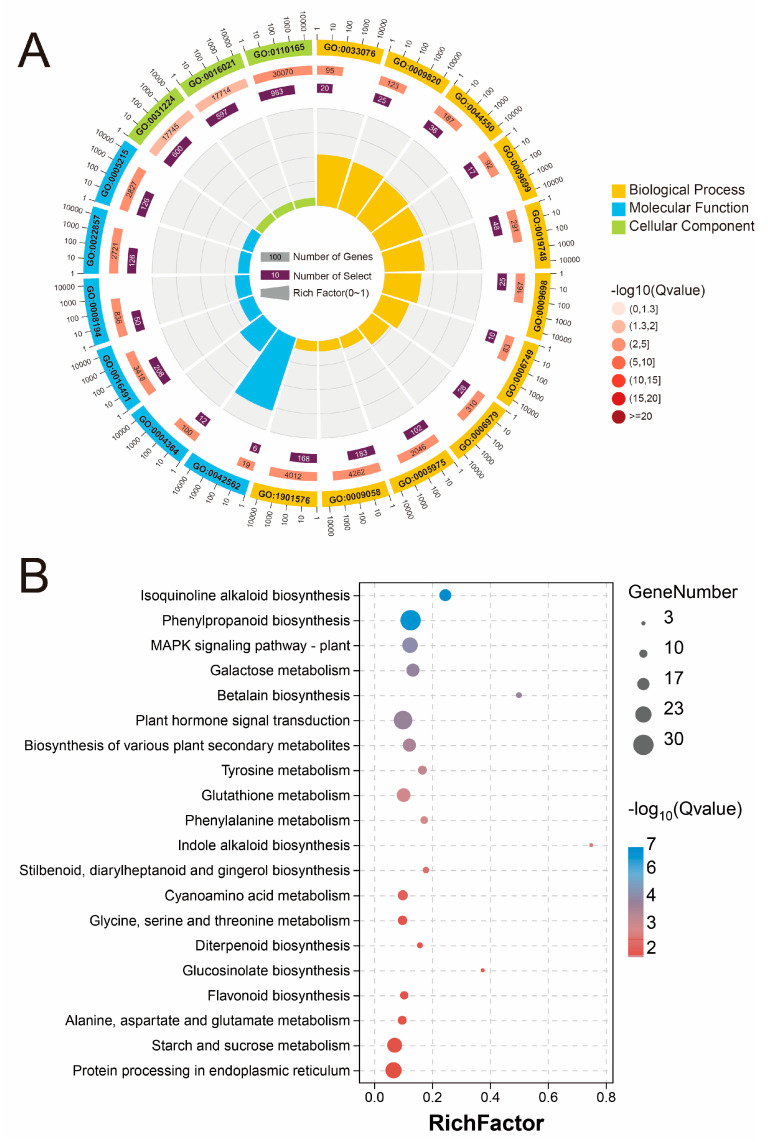
GO and KEGG enrichment analyses of DEGs in the NaCa vs. Na treatment group. (**A**) GO enrichment circle plot of the DEGs; (**B**) KEGG enrichment bubble chart of the DEGs.

The GO enrichment analysis revealed the GO terms associated with the different treatment groups, which were divided into three categories: biological processes (BPs), molecular functions (MFs), and cellular components (CCs) (Figure 4A and Figure 5A). The results revealed that responsive genes were enriched in “cellular anatomical entity”, “intrinsic component of membrane”, and “integral component of membrane” in the CC category. In the BP category, the top 10 GO terms were linked primarily to the synthesis and metabolism of secondary metabolites. In contrast, the enriched genes in the two groups were different in the MF category. Glutathione transferase activity, carbohydrate derivative transmembrane transporter activity, and polysaccharide binding were among the most significant GO terms in the MF group in the Na vs. control group, while in the NaCa vs. Na group, oxidoreductase activity, transmembrane transporter activity, and UDP-glycosyltransferase activity were the most significant.

The KEGG enrichment analysis revealed that the genes associated with the genes in the KEGG pathway set were differentially expressed among all the treatment groups (Figure 4B and Figure 5B). Glutathione metabolism (map00480) was the most significantly enriched KEGG pathway between the Na treatment group and the control group. Furthermore, genes related to plant hormone signal transduction (map04075), the MAPK signaling pathway (map04016), and several secondary metabolic pathways, such as isoquinoline alkaloid biosynthesis (map00950) and phenylpropanoid biosynthesis (map00940), were strongly enriched. The DEGs in the NaCa vs. Na group were highly enriched in secondary metabolism (flavonoid biosynthesis, map00941; phenylpropanoid biosynthesis, map00940; betalain biosynthesis, map00965), signal transduction (MAPK signaling pathway, map04016; plant hormone signal transduction, map04075), antioxidant metabolism (glutathione metabolism, map00480), and glucose metabolism (starch and sucrose metabolism, map00500; galactose metabolism, map00052).

In summary, the DEGs in each treatment group were primarily significantly enriched in secondary metabolism, glutathione metabolism, signaling hormone metabolism, glucose metabolism, and amino acid metabolism, suggesting that these may be the key pathways mediating salt stress relief in *M. wufengensis*.

### 2.6. Validation of RNA-Seq-Based DEGs in M. wufengensis Plants via qRT–PCR

**Figure 6:**
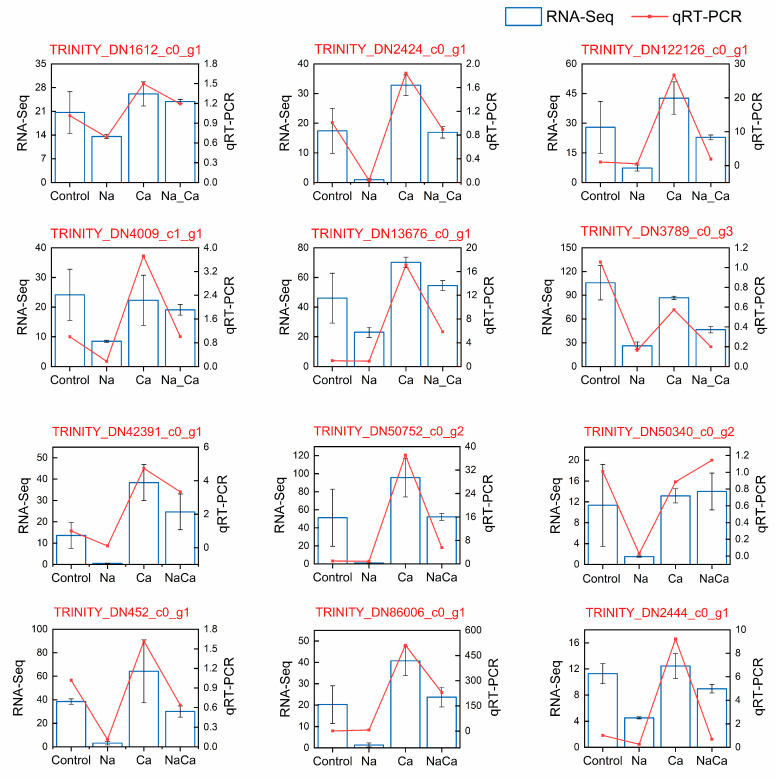
Verification via qRT–PCR of the expression of 12 randomly selected DEGs in *M. wufengensis* after four treatments.

The validation of the transcriptomic data (RNA-Seq data) in the present study was performed via qRT–PCR analysis. To confirm the reliability of the RNA-seq data, the relative expression patterns of 12 randomly selected DEGs were measured in the leaves of the *M. wufengensis*. The results showed that the trends in the two variables were basically the same (Figure 6), which indicates the reliability of the RNA-seq data.

### 2.7. Expression of Genes Involved in Transmembrane Transport

**Figure 7:**
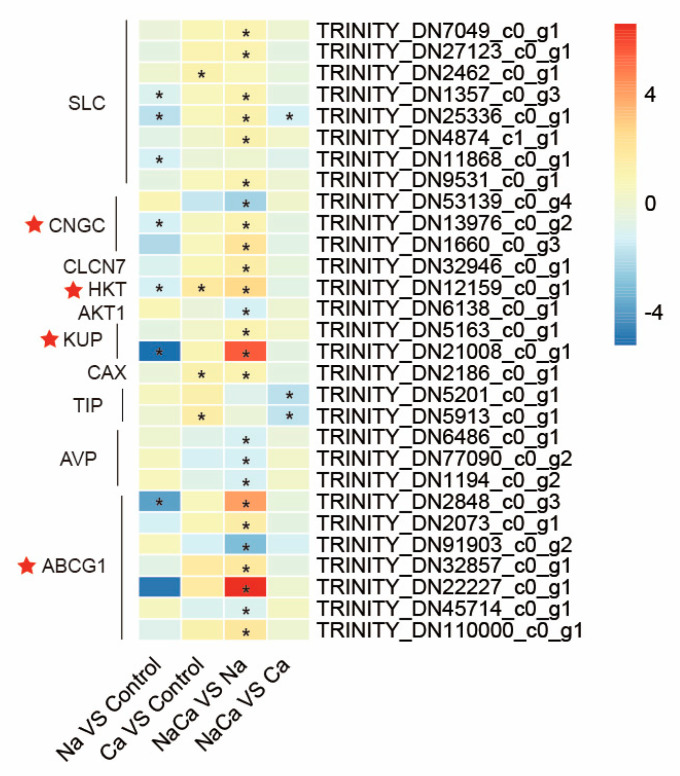
Differentially expressed genes associated with transmembrane transport. SLC: solute carrier gene; CNGC: cyclic nucleotide-gated channel gene; CLCN7: voltage-gated chloride channel 7 gene; HKT: high-affinity K^+^ transporter gene; AKT1: potassium channel protein gene; KUP: K^+^ uptake permease gene; CAX: cation/H^+^ exchanger antiporter gene; TIP: tonoplast intrinsic protein-encoding gene; AVP: pyrophosphate-energized vacuolar membrane proton pump gene; ABCG1: adenosine triphosphate (ATP)-binding cassette efflux transporter G1 gene. “*” indicates a significant difference in gene expression between the two treatments (*p* < 0.05).

The GO enrichment analysis confirmed that exogenous Ca^2+^ had significant effects on DEGs associated with transmembrane transport. The analysis of genes related to transmembrane transport revealed that 29 genes were differentially expressed, most of which were related to ion regulation (Figure 7 and Table S7). After Ca^2+^ addition, eight solute carrier (SLC) genes involved in multiple ion transmembrane exchanges were upregulated, and two cyclic nucleotide-gated channel (CNGC) genes regulating the Na^+^/Ca^2+^ balance were upregulated. Two K^+^ transport-related genes, a high-affinity K^+^ transporter gene (HKT), a K^+^ uptake permease gene (KUP), and one cation/H^+^ exchanger antiporter gene were upregulated. In addition, five adenosine triphosphate (ATP)-binding cassette efflux transporter G1 genes (ABCG1), which can help a cell to expel toxic substances and maintain the stability of its contents, were found to have an upregulated expression.

### 2.8. Identification of Plant Hormone and Signal Transduction-Related Unigenes

**Figure 8:**
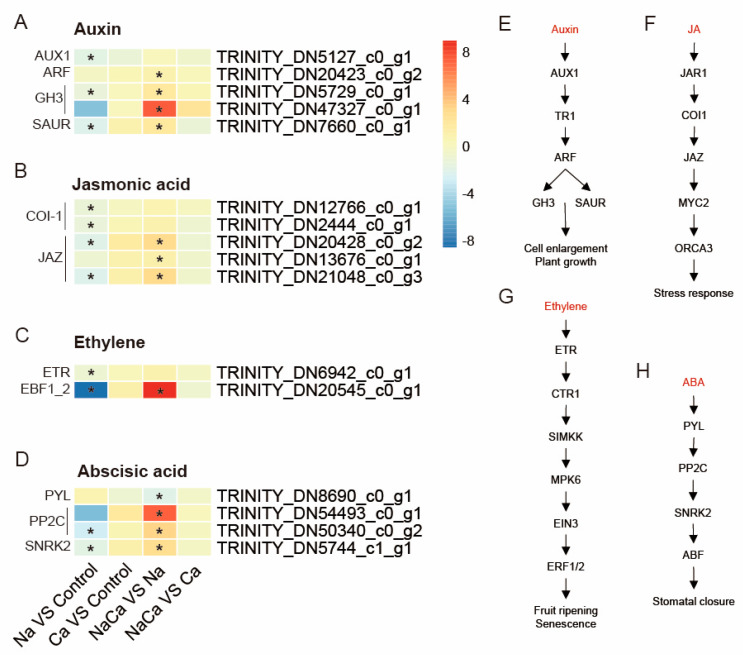
DEGs related to plant hormone signal transduction. (**A**–**D**) Heatmap analysis of auxin, jasmonic acid, ethylene, and abscisic acid-related DEGs; (**E**–**H**) auxin, jasmonic acid, ethylene, and abscisic acid signal transduction pathways. AUX1: auxin influx carrier; ARF: auxin response factor; GH3: auxin-responsive GH3 gene family; SAUR: small auxin-up RNA gene family; COI1: coronatine-insensitive 1; JAZ: jasmonate ZIM-domain gene; ETR: ethylene receptor gene; EBF1_2: EIN3-binding F-box protein 1 gene; PYL: abscisic acid receptor PYR/PYL family; PP2C: type 2C protein phosphatase; SNRK2: SNF1-related protein kinase 2. “*” indicates a significant difference in gene expression between the two treatments (*p* < 0.05).

Plant hormones are crucial substances for regulating plant growth and responses to stress. The GO and KEGG enrichment analyses revealed that DEGs involved in plant hormone signal transduction were significantly enriched. Therefore, we studied the expression of genes related to hormone signaling in the leaves of *M. wufengensis* (Figure 8). In this study, 32 DEGs were found to be involved in the regulation of hormones, including auxin (AUX), abscisic acid (ABA), brassinosteroids (BRs), cytokinin (CK), ethylene (Eth), gibberellin acid (GA), jasmonic acid (JA), and other related genes under different treatments (Table S8). To elucidate the effect of Ca^2+^ resistance on salt stress, we compared the expression of auxin, jasmonic acid, ethylene, and abscisic acid DEGs among the four treatments.

Under salt stress, the expression of the DEGs auxin resistance 1 (AUX1), auxin response factor (ARF), Gretchen Hagen3 (GH3), and small auxin-up RNA (SAUR) decreased. Compared with those under NaCl stress, the expression of the DEGs ARF, GH3, and SAUR was significantly upregulated under NaCa treatment (Figure 8A,E). These results suggest that Ca^2+^ treatment promotes auxin synthesis. In the JA signaling pathway, the expression of two COR-insensitive 1 (COI1) genes and two jasmonate ZIM-domain (JAZ) genes was downregulated, while that of three JAZ genes was upregulated under NaCa treatment compared with Na treatment (Figure 8B,F). In the ethylene signaling pathway, one ethylene receptor gene (ETR) and one EIN3-binding F-box protein 1 gene (EBF1_2) were downregulated under salt stress. Both genes were upregulated after exogenous Ca^2+^ treatment (Figure 8C,G). In the ABA signaling pathway, NaCl inhibited the expression of the type 2C protein phosphatase (PP2C) and SNF1-related protein kinase 2 (SnRK2) genes. Compared with those in the Na treatment group, the expression of the PP2C and SnRK2 genes in the NaCa treatment group was significantly upregulated (Figure 8D,H).

The DEGs identified in this study were significantly enriched in the MAPK signaling pathway (map04016), and a total of 24 DEGs of this pathway were found under the different treatments (Figure S2 and Table S8), among which 17 had the highest expression in the NaCa vs. Na treatment group. These genes were involved in four specific pathways, two of which are related to the hormone signal transduction pathways of ethylene and abscisic acid, which strongly overlap with the DEGs in the plant hormone signal transduction pathway mentioned above, indicating that these hormones play a role in activating the MAPK signaling pathway. In addition, DEGs were also distributed in the wounding and pathogen infection pathways. There were four CaM genes, one respiratory-burst oxidase (RBOH) gene, one LRR receptor-like serine/threonine protein kinase (FLS2) gene, two 1-aminocyclopropane-1-carboxylate synthase 6 (ACS6) genes, and two pathogenesis-related protein 1 (PR1) genes that were upregulated. These results indicate that exogenous Ca^2+^ plays a signaling role through Ca^2+^/calmodulin under salt stress.

### 2.9. Salt-Responsive Genes Related to Metabolism and Biosynthesis

**Figure 9:**
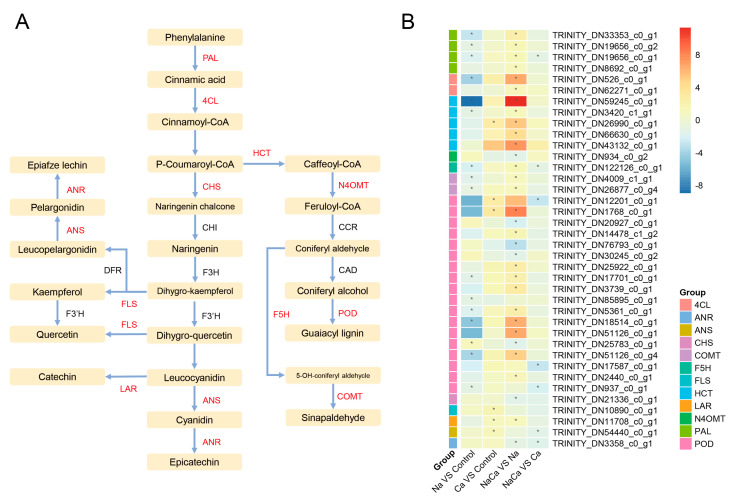
DEGs involved in the phenylpropanoid and flavonoid biosynthesis pathways. (**A**) Regulated pathways of DEGs related to the phenylpropanoid and flavonoid biosynthesis pathways; (**B**) heatmap of DEGs involved in the phenylpropanoid and flavonoid biosynthesis pathways. 4CL: 4-coumarate-CoA ligase; ANR: anthocyanidin reductase; ANS: anthocyanidin synthase; CHS: chalcone synthase; COMT: caffeic acid 3-O-methyltransferase; F5H: ferulate-5-hydroxylase; FLS: flavonol synthase; HCT: shikimate O-hydroxycinnamoyl transferase; LAR: leucoanthocyanidin reductase; N4OMT: norbelladine O-methyltransferase; PAL: phenylalanine ammonia-lyase; POD: peroxidase. “*” indicates a significant difference in gene expression between the two treatments (*p* < 0.05).

The biosynthesis of secondary metabolites was the most enriched category according to the GO and KEGG enrichment analyses. The phenylpropanoid and flavonoid biosynthesis pathways were the most highly enriched pathways, with 32 DEGs (Table S9). Here, we focused on the phenylpropanoid and flavonoid biosynthesis pathways in which a large number of DEGs were enriched (Figure 9). According to the results, there was 1 highly upregulated DEG and 15 highly downregulated DEGs involved in the phenylpropanoid and flavonoid biosynthesis pathways in the Na treatment group compared with the control group (Figure 9B). Moreover, the NaCa vs. Na group showed the opposite trend relative to that of the Na vs. control group, in which 25 highly upregulated DEGs and only 7 highly downregulated DEGs were found (Figure 9B). In addition, the Ca vs. control group exhibited a few upregulated DEGs, while the NaCa vs. Ca group exhibited a few downregulated DEGs, which may be related to the different effects of Na and Ca under salt stress. Figure 9A shows the total regulated pathways of flavonoid and phenylpropane biosynthesis. A total of 12 genes encoding PAL, 4CL, HCT, FLS, etc., which are the key enzymes involved in flavonoid and phenylpropane biosynthesis, were identified. The number of POD-related genes was the highest, followed by the number of HCT-related genes (18 and 5, respectively). Overall, NaCl treatment downregulated a large number of DEGs in the flavonoid and phenylpropanoid biosynthesis pathways in *M. wufengensis*, whereas Ca^2+^ promoted the synthesis of secondary metabolites, which may suggest an ameliorative effect of Ca^2+^ under salt stress.

**Figure 10:**
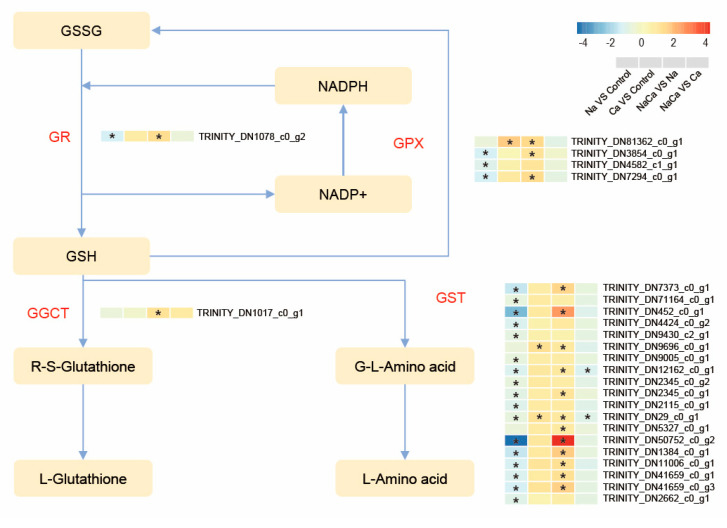
Heatmap and regulated pathway analysis of DEGs related to the glutathione metabolism pathway. GR: glutathione reductase; GPX: glutathione peroxidase; GST: glutathione S-transferase; GSH: glutathione; GSSG: oxidized glutathione. “*” indicates a significant difference in gene expression between the two treatments (*p* < 0.05).

The bioinformatics results revealed that one of the most highly enriched KEGG pathways was involved in glutathione metabolism (map00480). Among all of the treatment groups, 26 DEGs were commonly involved in glutathione metabolic pathways (Table S9). A high proportion (22 genes) of the genes were significantly downregulated in the Na treatment group compared to the control group. Most of the genes in both the Ca vs. control and NaCa vs. Na groups were upregulated, but the difference was more significant in the NaCa vs. Na group. Most of the genes in the NaCa treatment group were not significantly different from those in the Ca treatment group. These results suggest that genes related to GSH metabolism may play a key role in the mechanism of Ca^2+^ action in *M. wufengensis* under salt stress. There were 19 genes in the GST gene family and 4 genes in the GPX gene family among the genes in the glutathione metabolism pathway, and the rest were in the GGCT and GR gene families (Figure 10). As shown in the glutathione metabolic pathway diagram, Na treatment slowed the interconversion of GSH and GSSG and reduced GSH metabolism. However, after the addition of Ca^2+^, the expression of genes related to glutathione metabolism increased, which accelerated the expression of genes involved in this metabolic pathway.

In this study, a total of 16 DEGs were enriched in the starch and sucrose metabolic pathway (map00500) (Figure S3 and Table S9). In the Na vs. control treatment group, 13 genes were downregulated and 3 genes were upregulated, while in the NaCa vs. Na treatment group, 13 genes were highly upregulated and 3 genes were downregulated. Salt stress induced the expression of genes related to starch synthetase (glgA) and granule-bound starch synthase (WAXY), and promoted the synthesis of starch. Ca^2+^ addition induced the expression of sucrose synthase (SUS), trehalose 6-phosphate synthase/phosphatase (TPS), β-glucosidase (bglB), and other related genes, among which bglB genes were the most abundant and exhibited the most significant expression. The expression of genes related to starch synthesis was inhibited under NaCa treatment.

Both the NaCl and Ca^2+^ treatments significantly enriched genes related to amino acid metabolism, most of which were related to the metabolism of phenylalanine, cyanine, and tyrosine (Figure S4 and Table S9). NaCl treatment downregulated 8, 10, and 11 genes related to the metabolism of phenylalanine, cyanuric acid, and tyrosine, respectively, while Ca^2+^ treatment significantly upregulated 9, 11, and 10 genes related to these genes, respectively. Overall, salt stress significantly inhibited the metabolism of phenylalanine, cyanine, and tyrosine, while Ca^2+^ treatment significantly promoted their metabolism.

## 3. Discussion

Salinity is one of the most serious abiotic stresses worldwide and strongly affects plant growth and productivity [1]. Exogenous Ca^2+^ has previously been used to improve plant resistance to abiotic stress and has been found to improve salt tolerance by improving plant morphology and physiology [14,20,21]. Moreover, transcriptome sequencing has become a necessary method for studying the molecular mechanism of plant stress tolerance, and exploring the molecular mechanism by which Ca^2+^ improves plant salt tolerance through RNA sequencing has become increasingly important. In the present study, the underlying physiological and molecular mechanisms by which Ca^2+^ alleviates salt stress in the leaves of *M. wufengensis* were revealed through transcriptome sequencing.

The transcriptome data in the present study show that salt stress downregulated a large number of DEGs but upregulated only a few DEGs. NaCa treatment upregulated and downregulated abundant DEGs, which caused immediate stress reactions and activation of DEGs, suggesting the crucial role of exogenous Ca^2+^ in alleviating salt stress. Salt tolerance in plants is a complex physiological response that varies among different species, degrees of stress, and environments [22]. In this research, GO and KEGG enrichment analyses were used to classify DEGs under different treatments, and DEGs with significant differences were selected for further analysis to explore the mechanism by which exogenous Ca^2+^ alleviates salt stress in *M. wufengensis*.

### 3.1. Exogenous Ca^2+^ Maintained the Growth of M. wufengensis under Salt Stress

Previous studies have shown that the osmotic pressure difference between the inside and outside of the cell resulting from salt stress causes the cell to lose water and shrink, resulting in osmotic stress. When a great deal of Na enters the cell, the cell structure and membrane system are disrupted, oxidative stress occurs, and the operation and growth of the plant are affected by the ionic toxicity of Na^+^ [23]. In this study, the decreases in the leaf water content, plant growth rate, dry weight, and leaf chlorophyll content caused by Na^+^ further confirmed the negative effects of salinity on the growth of *M. wufengensis*. Exogenous Ca^2+^ can alleviate the symptoms of plant salt damage and restore growth; these findings have been verified in *Arabidopsis thaliana*, *Oryza sativa*, *Gossypium hirsutum*, and other plants [14,20,21]. In the present study, exogenous Ca^2+^ was able to alleviate salt stress symptoms, restore height and stem growth, and increase the plants’ dry weight, but it had no effect on the leaf water content, which may indicate that the mechanism by which Ca^2+^ improves salt tolerance may not be related to water homeostasis.

### 3.2. Exogenous Ca^2+^ Enhanced Plant Hormones and Signal Transduction in M. wufengensis under Salt Stress

In addition, plant hormones are key factors in the salt stress response and participate in the regulation of plant functions by activating signaling mechanisms. Ca^2+^ has been proven to interact with plant hormones to promote signaling and regulation under stress conditions. Dindas et al. [24] reported that Ca^2+^ is involved in regulating auxin signaling, primarily through the absorption of auxin by AUX1 transporters and the triggering of Ca^2+^ channels in CNGC14, resulting in Ca^2+^ inflow. The triggered Ca^2+^ signal can be transmitted over long distances to regulate the growth response. Moreover, these authors found that AUX1-mediated IAA transport was inhibited after the addition of Ca^2+^ channel blockers, suggesting that Ca^2+^ may exert a counterregulatory effect on the IAA signaling pathway. In the present study, the significantly increased expression of genes in the AUX1 pathway observed after Ca^2+^ addition is consistent with these findings. In the study by Liu et al. [16], the IAA concentration decreased under Na treatment and increased after Ca^2+^ addition, which is consistent with the results of the present study, indicating that Ca^2+^ participates in the transduction of IAA signaling under salt stress and promotes growth recovery.

Previous studies have shown that ABA is the major hormone regulating plant responses under salt stress and that regulating stomatal movement is an important strategy under salt stress [25,26]. ABA promoted by Ca^2+^ under salt stress can act on stomatal guard cells, induce stomatal closure, reduce transpiration, reduce water loss, and help plants alleviate damage caused by osmotic stress. ABA is usually produced in the above-ground parts of plants but acts heavily on the roots. Studies have shown that this long-distance transport may be related to Ca^2+^ signaling [25,27]. The expression of genes in the ABA hormone signaling pathway significantly increased in response to the addition of Ca^2+^, indicating that Ca^2+^ promotes ABA signal transduction under salt stress.

Like ABA, JA is involved in stomatal closure under salt stress. Förster et al. [28] reported that JA induces stomatal closure by activating a complex of calcineurin B-like proteins and CBL-interacting protein kinases (CBL1-CIPK5). Many studies have reported an increase in JA content under salt stress [29,30]. In the present study, the trends in the changes in the DEGs of JA and ABA were consistent, which may be related to the regulation of JA by ABA, as ABA accumulation can increase the JA content and enhance plant salt tolerance by regulating the repressor protein JAZ [31]. The results of the present study showed that Ca^2+^ was strongly involved in hormone signal transduction in *M. wufengensis* under salt stress, and the molecular mechanism through which Ca^2+^ interacts with plant hormone signaling molecules to regulate downstream genes requires further study.

The conversion of exogenous Ca^2+^ into a Ca signal after entering the plant is decoded by a calcium ion sensor. CaM is a major calcium ion sensor that regulates a wide range of target proteins, such as various ion channels, transcription factors, protein kinases, and protein phosphatases, and it can directly or indirectly regulate plant responses to environmental stress [32]. Studies have shown that ROS homeostasis and the entire antioxidant system of plants under stress conditions can be regulated by Ca^2+^ influx and Ca^2+^/CaM sensors, and CaM-mediated signals are also actively involved in plant responses to osmotic stress [32]. Overexpression of the *Glycine max* CaM gene in *Arabidopsis* can enhance its salt tolerance [33]. In this study, CaM, protein kinase, and protein phosphatase-related genes in the MAPK signaling pathway were significantly upregulated after Ca^2+^ addition, indicating that exogenous Ca^2+^ enhanced Ca^2+^ signal transduction to enhance the salt tolerance of *M. wufengensis*.

### 3.3. Exogenous Ca^2+^ Accelerated the Removal of Harmful Substances from M. wufengensis under Salt Stress

Our transcriptomic analysis revealed an increase in the expression of glutathione metabolism-related genes as Na^+^ and Ca^2+^ were added. GSH, an important antioxidant, has been shown to play a role in eliminating ROS under many abiotic stresses [34,35]. The action of GSH and ASA in the ASA-GSH cycle provides a highly efficient way to remove ROS [36]. In addition, the glutathione metabolism pathway is also a highly efficient ROS clearance system. In this study, the addition of Ca significantly increased the expression of most DEGs involved in the glutathione metabolism pathway, most of which were GST and GPX genes. GSH itself oxidizes to produce GSSG under the catalysis of GPX, and GSSH is reduced to GSH by GR, which can convert H_2_O_2_ into H_2_O [37]. In addition, GSH can also bind to the electrophilic groups of harmful substances via catalysis by GST, increasing its hydrophobicity and facilitating its discharge from the body to achieve detoxification. GST has dual functions in the detoxification and removal of lipid peroxides, although it does not participate in the decomposition of H_2_O_2_ [38]. These results suggest that exogenous Ca^2+^ supplementation can accelerate the glutathione metabolism pathway and help remove ROS and toxic substances to alleviate salt stress.

Our analysis of the transmembrane transport-related genes revealed that exogenous Ca^2+^ significantly upregulated the expression of five ABCG1 genes. ABCG genes belong to the ABC efflux transporter superfamily of genes involved in ATP-dependent transmembrane efflux of various molecules to facilitate normal physiological metabolism and responses to stress [39]. Studies on *Medicago sativa* have shown that the expression of the ABCG1 gene is responsive to NaCl stress [40]. Under the stress of heavy metals such as aluminum (Al), cadmium (Cd), and lanthanum (La), the ABCG1 gene has been found to be upregulated in *Axonopus compressus* [41]. The ABCG1 gene of *Hydrangea* was also found to be significantly enhanced under Al stress [42]. These results suggest that the ABCG1 gene responds to salt and heavy-metal stress at the transcriptional level, suggesting that one of its functions may be to transport metal ions. In this study, the upregulation of the ABCG1 gene after Ca^2+^ addition may have promoted the transportation of Na^+^ under salt stress, thus maintaining cell homeostasis in the leaves of *M. wufengensis*.

### 3.4. Exogenous Ca^2+^ Promoted the Equilibrium of the Osmotic System of M. wufengensis under Salt Stress

Plant secondary metabolites, including phenols, flavonoids, terpenoids, alkaloids, etc., are natural compounds with various physiological activities produced by plants through complex metabolic reactions and are primarily used as defense systems against various stress conditions [7]. Numerous studies have shown that appropriate stress stimulates the production of secondary metabolites, which are more prevalent in plants under biological stress, water stress, nitrogen stress, salt stress, and other stresses than in those under normal growth conditions [43]. However, the results of our study suggest that a large number of genes in the secondary metabolite biosynthesis pathway were downregulated under salt stress, possibly because the effects of salinity on plants vary according to species and variety, and the duration and degree of stress are also factors affecting the overall reactions of plants. The high degree and long duration of NaCl stress applied in this study may have been the causes of the reduced production of secondary metabolites under salt stress, which has also been observed in *Diplotaxis tenuifolia* and *Cynara scolymus* [44,45].

Secondary metabolites play a vital role in resisting salt stress. Secondary metabolites such as phenols and flavonoids act as important nonenzymatic antioxidants with rich antioxidant functions [43]. In this study, POD-related genes, which are the most abundant in the phenylpropanoid and flavonoid biosynthesis pathways, were found to be key regulators of plant antioxidant functions. In the regulated pathway, lignin produced by POD is the primary element in the secondary cell wall of plants [46]. Research has shown that plants can reduce the effects of ion stress by accumulating harmful ions in their cell walls [47]. Thickening of the cell wall has also been observed in salt-tolerant plants [48]. In this study, seven DEGs, including SOD, PAL, and HCT, were differentially upregulated after the addition of Ca^2+^, indicating that the synergistic expression of these enzymes and the lignification process enhances the tolerance of Magnolia Carthamus plants to NaCl. Research has shown that Ca^2+^ increases the expression of genes involved in secondary metabolite biosynthesis, which may be related to Ca^2+^-induced stomatal closure and a reduction in evapotranspiration [49], and the specific mechanisms behind this are worthy of further study.

The complexity of plant salt tolerance mechanisms is reflected in a variety of different cellular processes and metabolic pathways. Substances such as sucrose and amino acids are also involved in the plant stress response, which is conducive to maintaining plant osmotic balance [50]. Genes highly expressed under NaCa treatment were significantly enriched in the metabolic pathways of starch and sucrose, in which genes related to sucrose and trehalose synthesis were significantly upregulated while enzymes related to starch synthesis were significantly downregulated, indicating that the Ca^2+^ addition promoted the content of soluble sugars such as sucrose and trehalose and decreased the content of insoluble sugars such as starch. This trend is not only conducive to maintaining the osmotic balance of plants but also provides energy for plant growth under salt stress [23]. In addition, the expression of genes related to amino acid metabolism, such as phenylalanine, tyrosine, and cyanine, also increased with the addition of Ca^2+^ under salt stress. In salt-tolerant plants, amino acids not only play a role in maintaining osmotic balance but also regulate pH balance, stomatal conductance, protein content, and membrane stability [7]. These results suggest that exogenous Ca^2+^ plays an important role in maintaining the osmotic balance of *M. wufengensis* under salt stress.

### 3.5. Constructing a Hypothesis Model to Explain Ca^2+^-Regulated Salt Tolerance in M. wufengensis

**Figure 11:**
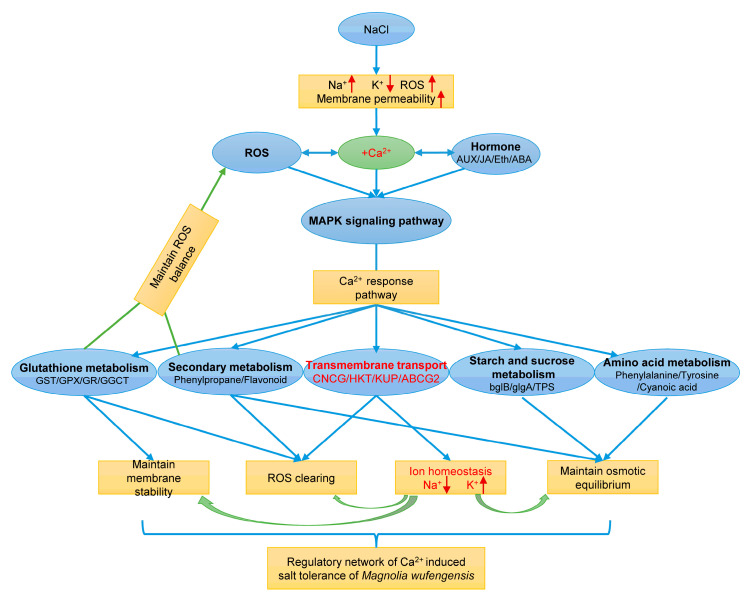
Hypothesis model showing Ca^2+^-mediated regulation of salt tolerance in *Magnolia wufengensis.*

Based on the above analysis, a hypothesis model of Ca^2+^-induced regulation of salt tolerance was constructed in this study (Figure 11). Salt stress caused an increase in Na^+^ and a decrease in K^+^ in *M. wufengensis* plants as well as an increase in ROS and membrane permeability. After the addition of Ca^2+^, Ca^2+^ signals are enhanced, Ca^2+^ signals interact with ROS and hormone signals to promote signal transduction jointly, and these signals are then primarily transmitted through the MAPK pathway. Ca^2+^ induced the response of genes related to transmembrane transport, enhancing the expression of CNGC, HKT, and other genes that maintain the Na^+^/K^+^ balance and ABCG1 and other toxin-clearing genes. Secondly, Ca^2+^ enhances the activity of the ROS-scavenging system, such as the activity of antioxidant enzymes such as GST, GPX, and POD, and further affects the transduction of ROS signals, maintaining the ROS balance in plants. Ca^2+^ also promotes the synthesis of phenylpropane, flavonoids, sucrose, and amino acids under salt stress, which further maintains homeostasis in plants. This hypothesis model reveals the potential molecular mechanism underlying the effect of Ca^2+^ on the salt stress response of *M. wufengensis*, identifies the key salt tolerance genes involved in related metabolic pathways, provides a theoretical basis for improving the salt tolerance of *M. wufengensis*, and provides a theoretical basis for breeding new salt-tolerant varieties of *M. wufengensis* via molecular breeding technology.

## 4. Materials and Methods

### 4.1. Plant Materials and Experimental Design

A Jiaohong 1 cultivar of grafted *M. wufengensis* seedlings provided by Wufeng Bo Ling Seed Industry Co., Ltd., from Wufeng, Hubei Province, was used in this study. The plants were grafted onto two-year-old *Yulania biondii* plants as rootstocks in March 2022. Healthy plants exhibiting uniform growth were planted in plastic pots (height: 320 mm; diameter: 360 mm) filled with 3.5 kg of turfy soil, with 1 plant per pot. The experiment was performed in June 2022 in a greenhouse with a mean temperature of 22/18 °C (day/night) in Beijing, China (40°3′54″ N, 116°05′45″ E).

A randomized complete block design employing two factors was used in this experiment. In the preliminary experiment, we initially subjected the *M. wufengensis* seedlings to varying concentrations of NaCl (soil NaCl content (weight ratio): 0‰, 1‰, 2‰, 3‰, and 4‰) and observed their ability to withstand NaCl stress, revealing that they could survive under 2‰ or less NaCl stress [50]. Then, the following experiment involved the use of four concentrations of NaCl (0‰, 2‰, 2.5‰, and 3‰) and four concentrations of CaCl_2_ (0, 1, 5, and 20 mM) to investigate the impact of CaCl_2_ on the plants under NaCl stress. The results showed that 5 mM CaCl_2_ had the best effect on plant growth at 2.5‰ NaCl stress, but had no effect under 3‰ NaCl stress (results unpublished). Therefore, two NaCl concentrations (0 and 2.5‰) and two CaCl_2_ concentrations (0 and 5 mM) were selected for transcriptome sequencing. There were four treatments in total in this experiment, each with 10 seedlings. The experiment was repeated 3 times. The specified concentrations of NaCl and CaCl_2_ were added to the soil according to the four treatments. As shown below, the total amounts of NaCl and CaCl_2_ added to each seedling in this experiment are listed in Table 3. To prevent the plants from dying because of the high-concentration treatments, the NaCl and Ca^2+^ treatments applied in this study were divided four times, each at intervals of 5 days, until the established concentrations were reached on the 15th day. After 60 days, the growth status of the seedlings was evaluated, and the mature leaves in the middle and top sections were collected, frozen in liquid nitrogen, and stored in a −80 °C freezer for RNA extraction and determination of physiological indices.

### 4.2. Determination of Plant Growth and Physiological Indices

To determine the relative growth rate (%), the height and stem diameter of *M. wufengensis* were measured at 0 d and 60 d.

After 60 days of treatment, 3 seedlings were randomly selected for each treatment, and cleaned with water. The seedlings were dried at 105 °C for 30 min and then at 65 °C for 48 h until their weight did not change.

The leaf water content (LWC) was calculated using the following formula:

LWC (%) = (fresh weight − dry weight)/(fresh weight) × 100(1)

The chlorophyll content was determined via ethanol extraction [50]. Fresh leaves (0.5 g) were ground with liquid nitrogen and immersed in ethanol (10 mL, 95%) for 24 h in a dark room. The absorbances were measured using a spectrophotometer (VERTEX70, Bruker, Billerica, MA, USA) and the chlorophyll contents were calculated.

The content of malondialdehyde (MDA) was determined using the thiobarbituric acid method [50]. Fresh leaves (0.5 g) were ground with 5 mL trichloroacetic acid (5%) at a low temperature and centrifuged at 10,000× *g* for 20 min (4 °C). Then, 2 mL supernatant was mixed with 2 mL thiobarbituric acid (0.6%), heated in a boiling water bath for 30 min, and centrifuged at 10,000× *g* again for 5 min (4 °C). The absorbance of the supernatant at 450, 532, and 600 nm was determined, and the content of MDA was calculated.

### 4.3. Transcriptome Sequencing

Twelve samples (from four treatments and three replicates) were subjected to full-length transcriptomic analysis. As a newly discovered species, the genome of *M. wufengensis* has not yet been sequenced, so we conducted a de novo transcriptome assembly and analysis in order to obtain more annotated genes that have been reported in other species. RNA-seq was performed according to the instructions provided by the equipment manufacturer (Invitrogen, Carlsbad, CA, USA), and genomic DNA was removed using DNase I. RNA quality was subsequently examined via gel electrophoresis. RNA sequencing and library construction were performed at Shanghai Majorbio Biopharm Technology Co., Ltd. (Shanghai, China), using an Illumina NovaSeq 6000 sequencer (Illumina, San Diego, CA, USA) according to the manufacturer’s instructions [51]. The original sequencing data were filtered using Fastp (https://github.com/OpenGene/fastp, accessed on 1 December 2023) in order to obtain high-quality sequencing data (clean data) for subsequent analysis. The clean data were subsequently subjected to de novo assembly with Trinity (https://github.com/trinityrnaseq/trinityrnaseq, accessed on 3 December 2023). The transcriptome sequences were subsequently matched with the following databases: the NR database (https://ftp.ncbi.nlm.nih.gov/blast/db, accessed on 13 December 2023), the NOG database (http://eggnog6.embl.de, accessed on 13 December 2023), the Swiss-Prot database (www.uniprot.org, accessed on 15 December 2023), the Pfam database (http://pfam.xfam.org, accessed on 15 December 2023), the GO database (http://geneontology.org, accessed on 16 December 2023), and the KEGG database (http://www.genome.jp/kegg, accessed on 16 December 2023).

### 4.4. Comparative Transcriptomic Analysis

Gene expression levels were measured using RSEM (https://github.com/deweylab/RSEM, accessed on 25 December 2023). The genes of the Na vs. control, Ca vs. control, NaCa vs. Na, and NaCa vs. Ca groups were compared and the differentially expressed genes (DEGs) were analyzed using DESeq2 with the screening conditions of |log2fold-change| > 1 and *p* value< 0.05 [52]. GO enrichment analysis was performed using Goatools (https://github.com/tanghaibao/Goatools, accessed on 28 December 2023) with the screening criterion of a *q* value< 0.05 to determine the enrichment pathways [53]. KEGG enrichment analysis was performed using KOBAS (http://kobas.cbi.pku.edu.cn/home.do, accessed on 28 December 2023), with the screening criterion of a *q* value < 0.05 to determine the enriched pathways [54].

### 4.5. Quantitative Reverse-Transcription PCR (qRT–PCR)

The RNA used for qRT–PCR was extracted using a plant RNA extraction kit (Kangwei Century Biotechnology Co., Ltd., Beijing, China) according to the manufacturer’s instructions. cDNA was reverse-transcribed from the RNA using an AccuRT Genomic DNA Removal Kit based on the manufacturer’s instructions (G592, Applied Biological Materials, Richmond, BC, Canada). To analyze the expression of the target genes after different treatments, RT–PCR was performed using SYBR Green Master Mix (ABI, Vernon, CA, USA). The qPCR primers used here were designed using Beacon Designer 7 (PREMIER Biosoft International, Palo Alto, CA, USA) (Table S1). *MwACTIN* was used as a reference gene for the analyses [55]. Each sample included three biological replicates and three technical replicates.

### 4.6. Statistical Analyses

SPSS Statistics (version 22.0, IBM, Armonk, NY, USA) was used to perform a two-way ANOVA, and Duncan’s multiple-comparisons test was used to evaluate comparisons between different treatments. The statistical data are presented as their means ± SDs. The significance level was considered to be *p* ≤ 0.05. Graphs were constructed using R Project (version 4.3.1, R Foundation for Statistical Computing, Vienna, Austria) and Origin Pro (version 2021, Origin Lab, Northampton, MA, USA). All of the physiological and transcriptomic experiments were repeated three times.

## 5. Conclusions

In conclusion, Ca^2+^ actively regulated the growth and relief of salt stress in *M. wufengensis*. The transcriptomic analysis revealed that the Ca^2+^ regulatory mechanism enhances salt tolerance under salt stress, as massive numbers of DEGs were activated under NaCa treatment, suggesting that Ca^2+^ may play multiple roles in salt stress. GO and KEGG enrichment analyses were performed to reveal the potential pathways associated with salt stress relief, and the results revealed abundant genes that were highly correlated with transmembrane transport, plant hormone and signal transduction, metabolism, and biosynthesis. These pathways were analyzed to screen key genes, revealing the ABCG1 genes, which are related to transmembrane transport; the CaM genes, which are related to signal transmission; and the GST, GPX, and POD genes, which are related to antioxidant enzymes. The results of this study reveal the potential molecular mechanism by which Ca^2+^ alleviates salt stress in *M. wufengensis* and provide a hypothetical salt tolerance model. These findings could lay the foundation for the mining and cloning of key genes that affect salt tolerance in *M. wufengensis* under salt stress.

## Figures and Tables

**Table 1:** Sequencing the *M. wufengensis* transcriptome in twelve leaf samples from the four treatment groups.

Sample	Clean Reads	Clean Bases	Error Rate (%)	Q20 (%)	Q30 (%)	GC Content (%)
Control_1	5.45 × 10^7^	8.03 × 10^9^	0.0251	98.10	93.98	47.44
Control_2	5.20 × 10^7^	7.69 × 10^9^	0.025	98.13	94.04	47.79
Control_3	7.29 × 10^7^	1.08 × 10^10^	0.0251	98.11	94.02	48.09
Na_1	5.38 × 10^7^	7.93 × 10^9^	0.0253	98.03	93.79	47.73
Na_2	4.48 × 10^7^	6.60 × 10^9^	0.0252	98.05	93.88	47.99
Na_3	6.22 × 10^7^	9.30 × 10^9^	0.0257	97.81	93.44	47.95
Ca_1	5.14 × 10^7^	7.67 × 10^9^	0.0253	97.93	93.82	48.18
Ca_2	4.86 × 10^7^	7.24 × 10^9^	0.025	98.09	94.07	47.62
Ca_3	4.97 × 10^7^	7.40 × 10^9^	0.0259	97.73	93.23	47.90
NaCa_1	6.00 × 10^7^	8.85 × 10^9^	0.0254	97.93	93.75	47.65
NaCa_2	5.77 × 10^7^	8.53 × 10^9^	0.0255	97.89	93.61	47.78
NaCa_3	5.69 × 10^7^	8.41 × 10^9^	0.0252	98.03	93.95	47.62

**Table 2:** Transcriptome sequencing sample assembly results.

Type	Unigenes
Total number	173,292
Total bases	123,213,932
Longest length (bp)	15,917
Shortest length (bp)	201
Average length (bp)	711.02
N50 length (bp)	1067
E90N50 length (bp)	2530
Fragment mapped percentage (%)	65.298
GC percentage (%)	42.04
TransRate score	0.28333
BUSCO score	C: 72.1% [S: 70.1%; D: 2.0%]

**Table 3:** Total amounts of solutions added to each seedling under NaCl and CaCl_2_ stress.

Treatment	Soil Weight (kg)	Soil NaCl Content (Weight Ratio)	NaCl Weight (g)	NaCl Solution Concentration (mM)	CaCl_2_ Weight (g)	CaCl_2_ Solution Concentration (mM)	Total Solution Volume (L)
Control	3.5	0‰	0	0	0	0	1
Na	3.5	2.5‰	8.75	150	0	0	1
Ca	3.5	0‰	0	0	0.56	5	1
NaCa	3.5	2.5‰	8.75	150	0.56	5	1

## Data Availability

The datasets generated or analyzed within this study are included in this published article and its additional files. All of the transcriptomic data from the 12 samples have been deposited in NCBI’s Sequence Read Archive (SRA) under accession number PRJNA1049594 (https://www.ncbi.nlm.nih.gov/bioproject/PRJNA1049594/, accessed on 24 August 2024).

## Supplementary Materials

The following supporting information can be downloaded at https://www.mdpi.com/article/10.3390/plants13172418/s1: Figure S1: Characteristics of unigenes. Figure S2: DEGs associated with the MAPK signaling pathway; Figure S3: DEGs related to starch and sucrose metabolism; Figure S4: DEGs related to amino acid metabolism; Table S1: List of primers; Table S2: List of frequency distribution of splice lengths; Table S3: Gene function annotations; Table S4: DEGs between each comparison; Table S5: GO enrichment analysis of DEGs; Table S6: KEGG enrichment analysis of DEGs; Table S7: List of genes involved in transmembrane transport; Table S8: List of genes involved in plant hormone and signal transduction; Table S9: List of genes involved in metabolism and biosynthesis.
