# Supplementary material for: Physiological and Transcriptomic Analyses Demonstrate the Ca2+-Mediated Alleviation of Salt Stress in Magnolia wufengensis
Source: Plants (Basel). 2024 Aug 29;13(17):2418. doi: 10.3390/plants13172418 (PMC11396891; doi:10.3390/plants13172418)
Supplement: Supplementary file 1 [file plants-13-02418-s001.zip › Supplementary Data.pdf]

Supplementary data

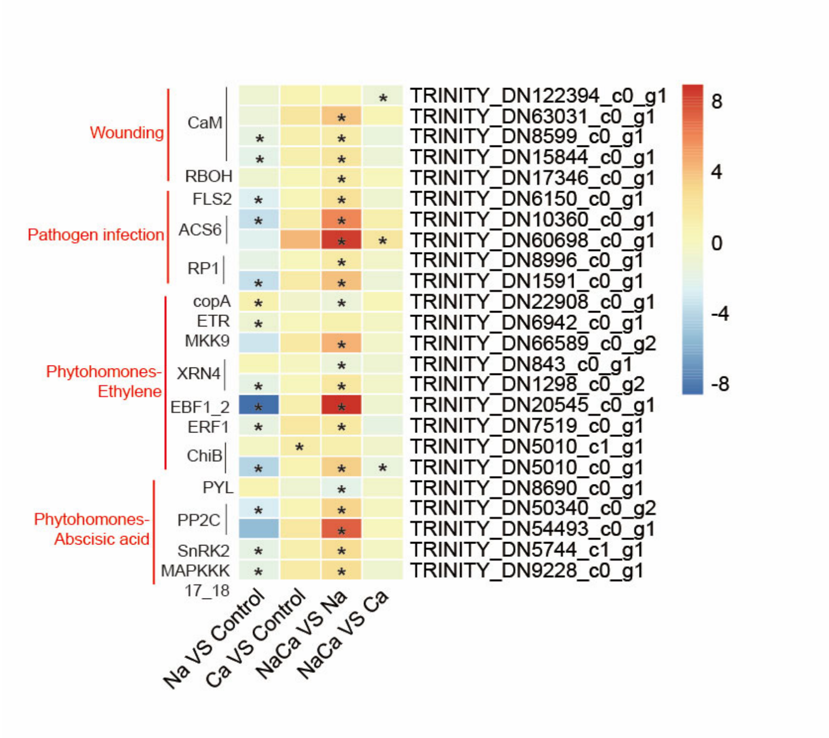

Figure S1 DEGs associated with MAPK signaling pathway. CaM: Calmodulin; RBOH: Respiratory burst oxidase; FLS2: LRR receptor-like serine/threonine-protein kinase; ACS6: 1-aminocyclopropane-1-carboxylate synthase 6; PR1: pathogenesis-related protein 1; copA: P-type Cu+ transporter; ETR: ethylene receptor; MKK9: mitogen-activated protein kinase kinase 9; XRN6: 5'-3' exoribonuclease 6; EBF1_2: EIN3-binding F-box protein; ERF1: ethylene-responsive transcription factor 1; ChiB: basic endochitinase B; PYL: abscisic acid receptor PYR/PYL family; PP2C: type 2C protein phosphatase; SNRK2: SNF1-related protein kinase 2; MAPKKK: mitogen-activated protein kinase kinase kinase 17/18.

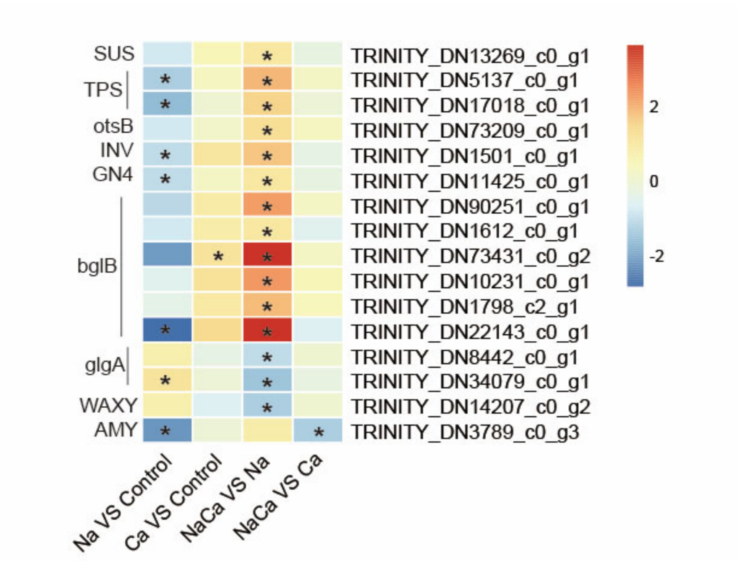

Figure S2 DEGs related to starch and sucrose metabolism. SUS: sucrose synthase; TPS: trehalose 6-phosphate synthase/phosphatase; otsB: trehalose 6-phosphate phosphatase; INV: beta fructofuranosidase; GN4: glucan endo-1,3-beta-glucosidase 4; bglB: beta-glucosidase; glgA: starch synthase; WAXY: granule-bound starch synthase; AMY: alpha amylase.

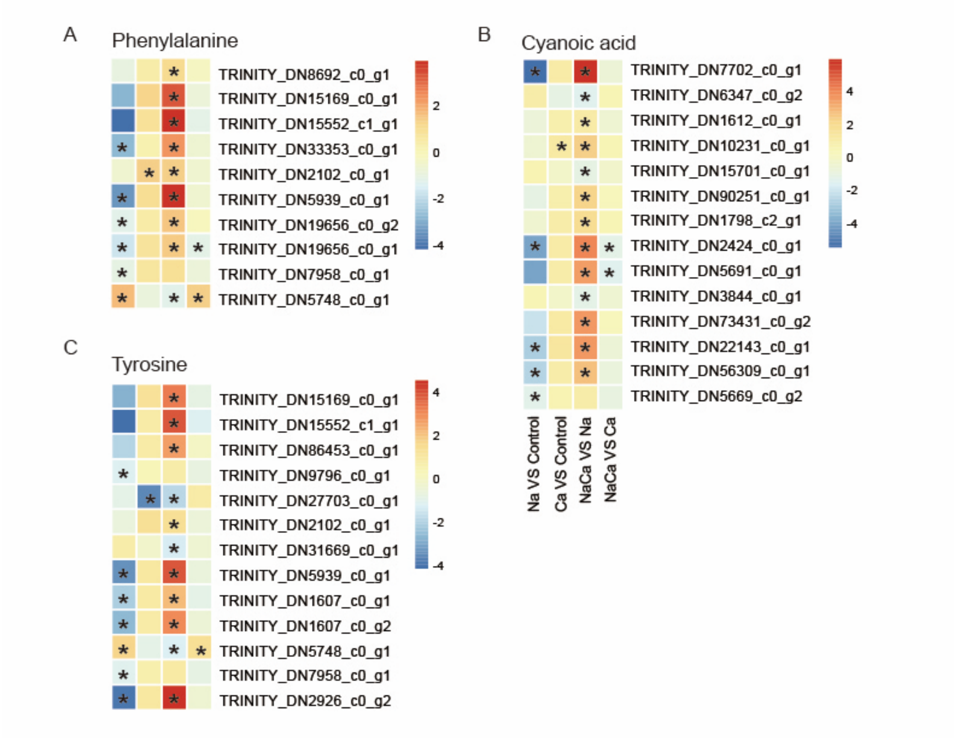

Figure S3 DEGs related to amino acid metabolism. (A) Phenylalanine; (B) Cyanoic acid; (C) Tyrosine.
